# Diversity in United States Dementia Prevention Trials: An Updated Systematic Review of Eligibility Criteria and Recruitment Strategies

**DOI:** 10.1159/000543905

**Published:** 2025-02-13

**Authors:** Najoua Lazaar, Sabine E. Van Beek, Awaale F. Rirash, Janne M. Papma, Jaime Perales-Puchalt, Ashley R. Shaw, Eric D. Vidoni, Sanne Franzen

**Affiliations:** a Department of Neurology and Alzheimer Center, Erasmus MC University Medical Center, Rotterdam, The Netherlands; b Department of Geriatrics and Alzheimer Center, Erasmus MC University Medical Center, Rotterdam, The Netherlands; c Department of Neurology, University of Kansas Alzheimer’s Disease Research Center, University of Kansas Medical Center, Kansas City, KS, USA

**Keywords:** Randomized controlled trials, Cultural diversity, Clinical trial protocols, Ethnic groups, Patient recruitment

## Abstract

**Introduction:**

Given the elevated dementia risk in underrepresented demographic groups in the USA – particularly in Latino and Non-Latino Black individuals compared to Non-Latino White individuals – it is vital that these groups are well-represented in dementia prevention research. Eligibility criteria and recruitment strategies may play a key role in promoting participant diversity. The aim of this review was to examine eligibility criteria and recruitment strategies in US dementia prevention trials in light of participant diversity.

**Methods:**

A systematic review was conducted using Medline (including PubMed), Embase, Cochrane Library, and CINAHL. We explored the percent White participants for trials using versus not using a specific eligibility criterion or recruitment strategy using Hodges-Lehmann median difference estimation.

**Results:**

Of forty-four studies meeting the inclusion criteria, twenty-seven reported on racial/ethnic diversity. Analyses demonstrated that criteria regarding cardiovascular disease, pulmonary disease, hearing impairment, and sedentary lifestyle were associated with relatively high participant diversity, while gastro-intestinal/liver disease, motivation to participate, and language proficiency criteria were associated with relatively little diversity. Information on recruitment strategies was often lacking. Three studies described recruitment efforts explicitly aimed at increasing diversity. Recruitment strategies associated with relatively high racial/ethnic diversity included recruitment via referral/word-of-mouth, television/radio advertising, and recruitment at church.

**Conclusion:**

Eligibility criteria could be improved by revisiting and revising how they are defined (e.g., motivation to participate). Regarding recruitment, several recommendations are provided, including (1) lifting barriers to study participation (e.g., through reimbursement), (2) collaborating with community partners, and (3) formally studying the effectiveness of recruitment strategies.

## Introduction

In the USA 6.7 million people have Alzheimer’s disease (AD) dementia [1]. Latino and Non-Latino (NL) Black/African American individuals are 1.5–3 times more likely to develop dementia compared to NL White individuals [1–4]. Various interacting factors may contribute to these disparities, including physical risk factors (e.g., vascular comorbidities [5]) social/structural determinants of health – such as socioeconomic status, employment, living environment, and quality of health care [5–7] – and macro level influences, such as structural racism and discrimination [8]. Several risk factors are modifiable through interventions and can thus play a key role in reducing dementia incidence among diverse populations [9]. To ensure equal effectiveness, safety, and feasibility of these interventions, it is essential that those most at risk – Latino and NL Black/African American individuals – are included. However, inclusion of diverse groups remains low, with only 11% non-White and Latino participants in dementia research in general [10] and 5% in AD drug trials [11]. Additionally, data on dementia prevention are scarce, as only 62% of US dementia prevention trials reported participants’ race/ethnicity. In these studies, 26% of the participants were ethnically and racially minoritized individuals [12].

Barriers to participation in AD research occur at micro, meso, and macro levels [13]. The micro level covers personal factors such as limited research literacy and mistrust of academic institutions [14], which often stem from macro level influences including historical injustice in scientific research and current discrimination in health care. Two important meso level barriers include trial eligibility criteria and recruitment strategies. A review of AD drug trials [11] highlighted that individuals from underrepresented groups may be disproportionately affected by eligibility criteria regarding medical conditions like diabetes, cardiovascular and cerebrovascular disease, renal disease and major psychiatric conditions. Furthermore, Raman et al. [15] showed that using cognitive outcomes like the Clinical Dementia Rating (CDR) or the Mini-Mental State Examination (MMSE) in AD trials can lead to a disproportionate exclusion of Black and Latino participants. Recommendations include using alternative cognitive tests and clearer definitions, reaching consensus regarding exclusion based on medical conditions, and removing procedural and linguistic barriers [11]. Few studies examine eligibility criteria in dementia prevention trials. One study [16] demonstrated that applying the criteria used in multidomain dementia prevention trials to a community-based sample (*n* = 5,381) resulted in a failure to include those at highest risk of developing dementia and led to an overinclusion of participants already qualifying for routine cardiovascular interventions. The ethnoracial diversity of the study sample was limited, however, precluding analyses addressing the impact of the eligibility criteria on racial/ethnic diversity.

Another meso-level barrier that may impact participant racial/ethnic diversity is recruitment strategies [15, 17], such as the recruitment materials used, recruitment location, or composition of the research team. Using interpreters, translating study materials or employing bilingual and bicultural researchers may be helpful [18, 19], as well as adjusting the means of communication or location of recruitment [17]. Formal scientific evidence is lacking, however, on the exact effectiveness of each of these strategies [17].

No studies to date have assessed the potential impact of dementia prevention trials’ eligibility criteria and recruitment strategies on racial/ethnic diversity. The aims of this study were therefore to examine which criteria and recruitment strategies are used in dementia prevention studies, how they are operationalized and reported, and how these criteria and strategies may impact participant racial/ethnic diversity levels. To this end, we will report and compare racial/ethnic diversity levels in prevention trials per eligibility criterion and per recruitment strategy. Furthermore, we will examine instances where the clarity/specificity and operationalization of criteria could be improved to prevent inadvertent exclusion of ethnoracially diverse populations.

## Methods

### Search Strategy, Title/Abstract Screening, and Data Extraction

Shaw et al. [12] previously systematically reviewed participant racial/ethnic diversity in phase II and III US-based dementia prevention randomized controlled trials (RCTs). We updated the search up to April 21st, 2023. Titles and abstracts of newly identified records were screened by NL and SvB, who also extracted eligibility criteria and recruitment strategies. All available sources were used: scientific publications, the Cochrane Library, and registrations in clinicaltrials.gov. We did not evaluate the methodological quality of the included studies, as the effect of the intervention itself was not studied in this paper.

### Eligibility Criteria for Studies in This Review

We utilized the same eligibility criteria for article selection as outlined in the previously published paper [12].

#### Inclusion Criteria

1. Phase II and III randomized controlled trials;
2. Enrollees with normal cognition and aged ≥45 years old;
3. Minimum of 1 explicitly identified cognitive outcome measure;
4. USA-based trials.

#### Exclusion Criteria

1. Investigational medication trials seeking FDA-approval;
2. Trials aimed at treatment of existing cognitive impairment;
3. Psychiatric-related cognitive trials (i.e., major depression as primary diagnosis);
4. Protocol-related articles;
5. Clinical trials that did not provide results;
6. Randomized controlled trials with no cognitive outcome examined;
7. Retrospective articles;
8. Secondary articles.

### Analyses

Operationalizations of the eligibility criteria were examined and described in a narrative way, highlighting instances where criteria and their cut-offs varied substantially across studies and cases in which the operationalization lacked specificity. To quantitatively examine the overall level of diversity, estimated pooled representation and confidence intervals (CIs) were calculated using R 4.4.0 and the metaprop function from the “meta” package in R. To examine the association between racial/ethnic diversity and each individual eligibility criterion and recruitment strategy, we first calculated the median percent White participants for trials using versus not using a specific eligibility criterion or recruitment strategy. We then compared these median percentages by estimating Hodges-Lehmann median differences, with 95% CIs. As sample sizes were small for some eligibility criteria and racial/ethnic diversity data was often not reported, this was considered a more suitable way of comparing racial/ethnic diversity levels than reporting Mann-Whitney U tests with *p* values. Criteria with insufficient data on race/ethnicity were excluded from the analysis (operationalized as less than four studies). For example, only two studies with a “computer use” eligibility criterion reported race/ethnicity data, precluding any meaningful analysis of the possible impact of this criterion on racial/ethnic diversity.

## Results

We will first discuss general findings on participant diversity and provide a comparison of the racial/ethnic diversity for trials that did versus that did not use a specific eligibility criterion. We will then illustrate how different types of eligibility criteria were defined, to highlight possible areas for improvement. We will discuss criteria regarding (1) medical conditions, (2) cognition and language, (3) sedentary lifestyle and diet, and (4) criteria relating to participation. We subsequently examine racial/ethnic diversity across the different recruitment strategies and illustrate the types of recruitment strategies used.

### Overview of the Included Studies

**Fig. 1.**
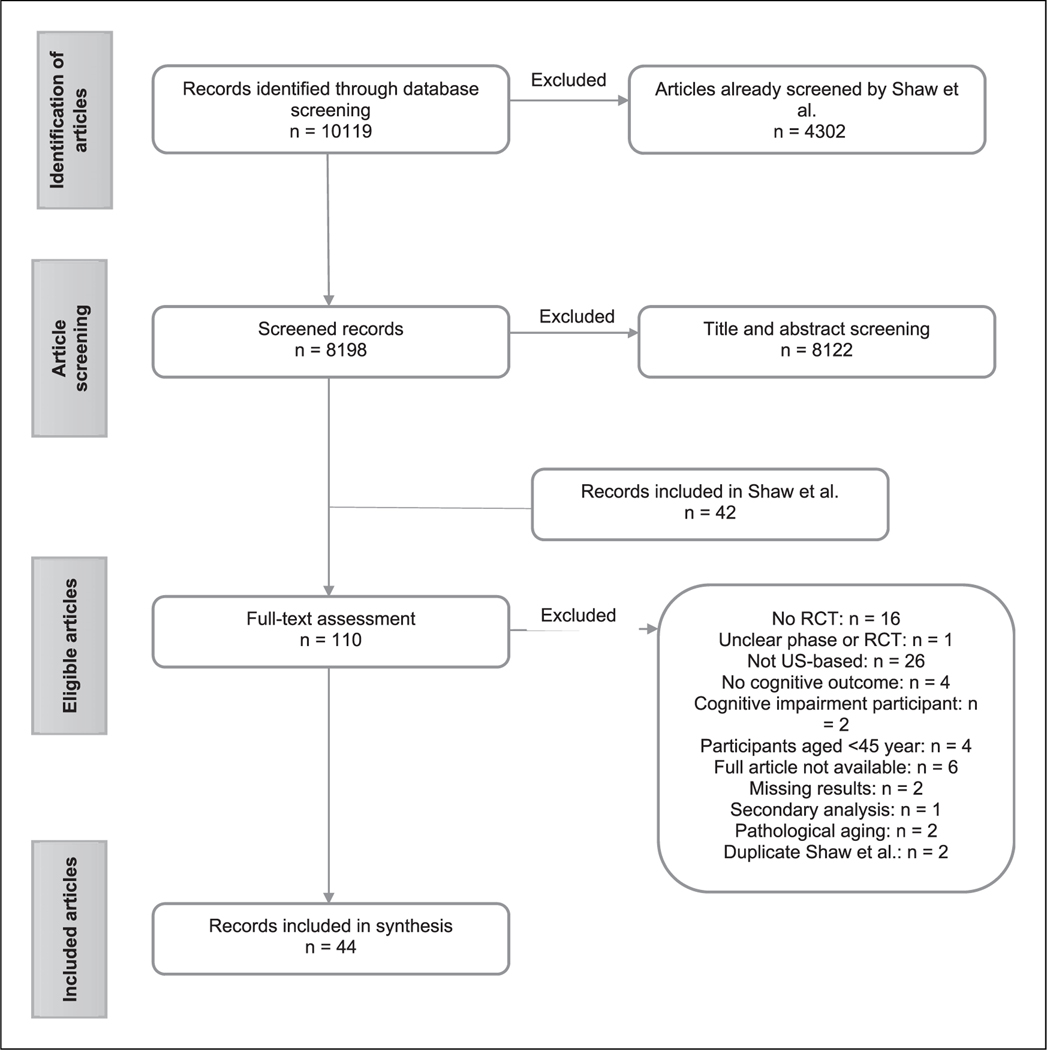
Database results and article selection.

Of the 11,019 records retrieved, 110 underwent full-text screening and 44 were included (see Fig. 1 for a PRISMA [20] flowchart and online suppl. Table 1; for all online suppl. material, see https://doi.org/10.1159/000543905 for a list of included studies). Interventions mainly included supplements (16/44, 36%), exercise (14/44, 31%), cognitive training (8/44, 18%), nutrition/diet (5/44, 11%) exergaming (2/44, 5%), dancing (1/44, 2%) and yoga (1/44, 2%), or a combination (3/44, 7%). One study specifically used Latin dancing to enroll Latino participants, as it was previously demonstrated one of the “only age-appropriate forms of physical activity in older Latina women,” with demonstrated feasibility [21]. Notably, multidomain trials were also included, combining interventions targeting multiple areas, such as cognitive therapy and dance lessons [22], cognitive training with physical activities [23], and physical activity with dietary modifications [24].

### Racial/Ethnic Diversity among Participants in Prevention Trials

In the original review, the pooled percentage of ethnoracially minoritized participants was 26%; one new study had 97% NL White participants [25], while the other study did not report on racial/ethnic diversity [26]. The pooled percentage in the updated review was 75.4% (CI: 61.7%; 87.0%) for White participants, 4.9% (CI: 1.3%; 10.4%) for Black participants, 0.3% (CI: 0.0%; 0.8%) for Asian participants, 0.00% (CI: 0.0%; 0.0%) for American Indian/American Native participants, 0.0% (CI: 0.0%; 0.0%) for Native Hawaiian and Pacific Islander participants and 3.8% (CI: 1.7%; 6.6%) for participants with another or unspecified background. A pooled percentage of 4.1% (0.1%; 12.2%) Latino participants was included. These proportions are not representative of the US population, which consists of 13.7% people who identify as Black (alone) 6.4% who identify as Asian (alone), 1.3% who identify as American Indian/Alaska Native (alone), and 0.3% who identify as Native Hawaiian and Pacific Islander (alone) – in addition to the 3.1% of the population identifying as biracial or multiracial and 58.4% identifying as NL White (with 75.3% identifying as White in total) [27]. Of the studies reporting main reasons for exclusion (12/44, 71%), none specified this data by racial/ethnic diversity. To nonetheless examine the potential impact of eligibility criteria on participant racial/ethnic diversity, we compared the median %NL White participants for trials that did vs. did not use a specific eligibility criterion (see Table 1). Due to the inadequate reporting of racial/ethnic diversity, analyses should be seen as exploratory. Differences (Hodges-Lehmann estimator) should be interpreted as follows: an interval that does not include zero is likely statistically significant and a wide interval indicates a less precise estimate. For interested readers, results from Mann-Whitney U tests (including *p* values) are available in online supplementary Table 2.

Cardiovascular and pulmonary disease, as well as hearing impairment, were the only eligibility criteria for which the entire CI was above zero, indicating a substantial and likely significant difference. Participant diversity was also relatively high in trials with a sedentary lifestyle eligibility criterion. None of the eligibility criteria had a CI fully below zero; however, trials with a criterion regarding gastro-intestinal/liver disease, supplement use, language proficiency, and motivation to participate had somewhat lower participant diversity. We will now provide an overview of how the eligibility criteria were defined.

### Operationalization of the Eligibility Criteria

#### Medical Conditions

Commonly used eligibility criteria for safety and to prevent inclusion of individuals with non-AD cognitive impairment included neurological conditions (28/44, 64%), diabetes (18/44, 41%), cardiovascular disease (27/44, 61%), cancer/malignancy (20/44, 45%), and renal disease (14/44, 32%). A small number of studies specifically included participants with these conditions (e.g., those with hypertension in an antihypertensive trial). The eligibility criteria varied widely in specificity, with some specifying metrics, while others, e.g., excluded participants with an “illness that requires >1 visit per month to a clinician.”

Five studies [23, 28–31] required some form of approval from the participant’s physician, such as preapproval or a health status report. One study [22] described using a screening tool to identify conditions that could preclude exercise participation, including a recommendation for when physician evaluation is needed.

#### Cognition and Language

Of the trials using a cognitive eligibility criterion, the majority used a cut-off on a specified cognitive or informant-reported instrument (18/44, 41%), such as the MMSE or the CDR. Many studies exclusively enrolled participants proficient in English (16/44, 36%). Five studies [23, 25, 32–34] included an eligibility criterion regarding (an absence of) self-reported cognitive decline or memory complaints. One study [35] included a list of seven questions, touching upon both proficiency and cognitive functioning (see online suppl. Box 1).

#### Sedentary Lifestyle and Diet

Fifteen studies (34%) only included participant with a sedentary lifestyle. Four studies [31, 36–38] stated “sedentary lifestyle” without further specification; two [39, 40] used a scoring model; nine [22, 23, 28, 29, 41–45] used exercise frequency, ranging from <3x per week to <1x per 13 weeks. Six studies combined the frequency with a time limit [23, 29, 41, 42, 44, 45], ranging from <20 min to <210 min per week. Two studies [39, 43] used the description of the American Heart Association standards for average fitness. Three studies excluded individuals based on their diet (vegetarian [34]/vegan [34, 46]). Some criteria were specific to a subset of the trials; for example, only trials investigating supplements had a supplement use eligibility criterion, while an eligibility criterion related to sedentary lifestyle was common in physical training interventions (10/14 studies) but rare in studies of supplements (1/16 studies).

#### Eligibility Criteria Relating to Participation

Fifteen studies (34%) described “motivation” as an eligibility criterion. Six defined this as being motivated to use or participate in the intervention when randomized in this group [23, 40–42, 47, 48], while eight described it as being motivated for follow-up or evaluation moments during the trial [26, 35, 40, 47, 49–52]. Three articles characterized motivation as willingness to forego use of supplements or medication other than those used in the study [25, 53, 54]. Another example of criteria related to participation included investigator’s doubts about therapy compliance [53–55].

### Racial/Ethnic Diversity and Recruitment Strategies

Regarding recruitment strategies, several different forms of communication and advertisement were used, particularly mailings (18/44, 41%) and newspaper advertisements (11/44, 25%). The level of detail in reporting varied considerably. Four studies [46, 50, 56, 57] did not define their recruitment methods. Of the ten studies (22%) reporting how many individuals were approached and screened, none reported enrollment rates per strategy. Details about which participants were contacted by researchers via mail, how they were approached, or the language in which the mailings or advertisements were written was generally lacking.

Table 2 presents the median %NL White participants for studies that did versus did not use a specific recruitment strategy (see online suppl. Table 3 for Mann-Whitney U tests with *p* values). Although none of the CIs fell fully above or fully below zero, trials recruiting through referrals/word-of-mouth, television/radio advertising, and at the church showed a relatively high level of diversity.

Two studies [22, 41] exclusively enrolled Latino individuals, of which one [22] reported recruiting in an area with a large Latino population. Another study aimed to recruit a “diverse sample of older adults” [58], using “state driver’s license and identification card registries, medical clinic rosters, senior center and community organization rosters, senior housing sites, local churches, and rosters of assistance and service programs for low-income elderly persons.” No other study reported enrollment strategies targeting a specific demographic group.

Regarding inclusion through referral and word-of-mouth, five studies used physicians referrals [23, 38, 43, 45, 59], while one also reported word-of-mouth recruitment via friends [23] and one via university offices [60]. Furthermore, snowball sampling was implemented within specific communities [31, 42, 51], a senior center [31] and amongst personal connections of the lead investigator [22]. Multiple studies reported recruiting in communities, including at community-oriented events like presentations in nursing homes, attending of job markets, information booths near churches and in health-care centers, community talks, and community-based screenings. It was not reported what specific strategies were used to approach these communities, liaise with community representatives, and build trust.

Eight studies (18%) reported recruitment in a specific location, of which seven recruited near the university or study site [23, 25, 30, 45, 48, 59, 60]. Regarding recruitment from databases, research-related databases were used most frequently (9/44 studies, 20%). Some studies used health care-related databases [55, 61] or municipality databases of voter-registration data [48].

## Discussion

The systematic review aimed to examine how eligibility criteria and recruitment strategies impact participant diversity in US dementia prevention trials. An overview of main findings and recommendations is provided in Table 3. Analyses of studies reporting on racial/ethnic diversity indicated that trials with eligibility criteria for cardiovascular disease, pulmonary disease, hearing impairment, as well as sedentary lifestyle showed relatively high diversity, while somewhat lower participant diversity was observed for trials with eligibility criteria relating to gastro-intestinal/liver disease, supplement use, language proficiency, and participants’ motivation. Relatively high racial/ethnic diversity levels were observed for trials recruiting via referral/word-of-mouth, television and radio advertising, and recruitment at the church.

Trials using a cardiovascular (exclusion) criterion were more diverse than those not using such a criterion. The prevalence of cardiovascular disease varies, however, across racial/ethnic diversity and different forms of cardiovascular disease. Hypertension is more prevalent in NL Black than NL White populations, with the lowest rates for Latino individuals [65], while rates of all-cause cardiovascular disease are highest in NL White individuals, followed by NL Black and Latino populations. The influence of cardiovascular eligibility criteria may thus depend on the interaction of race, ethnicity, and environment, as well as the specific cardiovascular condition. Similarly, prevalence rates for pulmonary conditions also vary per condition, generally with highest rates in NL White or NL Black populations, and lowest rates in Latino populations [65]. Likewise, varying prevalence rates exist for hearing impairment/loss, another eligibility criterion associated with more racial/ethnic diversity (although sample size was small). Overall, evidence suggests that NL Black and White individuals have the highest hearing loss prevalence, but estimates are extremely variable (e.g., 3.9%–91.8% for NL White individuals and 2.3%–48.1% in Latino populations [66]).

One of the most common eligibility criteria specific to dementia prevention trials was sedentary lifestyle. As it was often not clearly defined, we recommend researchers to properly define sedentary lifestyle in future studies. The trials including participants with sedentary lifestyles had relatively high racial/ethnic diversity levels. This is in line with the prevalence rates of sedentary lifestyle, as the highest rates of physical inactivity are observed within Latino (32%), NL Black (30%), and NL American Indian/Alaska Native (29%) communities, as compared to NL White communities (23%) [67]. These elevated rates of sedentary lifestyle and other risk factors for dementia are in turn intricately linked with social determinants of health [68–70]. For example, more leisure time physical activity is associated with high levels of education [71], although a trade-off may exist with occupational physical activity in some populations [72]. This association between education and physical activity is particularly relevant when considering racial and ethnic disparities, as historical and systemic inequities have resulted in unequal access to educational opportunities for certain racial and ethnic groups, which subsequently impacts health behaviors like physical activity and health outcomes [73–75]. Members of communities with limited access to education may not be as aware of the health risks of sedentary behavior or lack resources to engage in physical activity [76, 77]. These disparities can subsequently contribute to higher rates of chronic diseases related to sedentary lifestyles, such as obesity, diabetes, cardiovascular disease, and dementia among these populations [78].

Another common eligibility criterion was investigators’ perceptions of participants’ level of motivation (e.g., to participate in follow-ups or evaluation moments) or likelihood of completing the trial. Although estimates did not show clear associations of racial/ethnic diversity with this eligibility criterion, there is reason to believe it might disproportionately affect underrepresented populations, e.g., when an investigator assumes that a participant has a limited level of health literacy [79] or when physicians do not refer due to (1) costs to patients, (2) fear of losing patients due to mistrust, (3) cultural barriers, (4) an assumed lack of interest, (5) limited access to the study site, and (6) complex informed consent procedures [80].

A small number of studies required participants to obtain approval and/or a health status report from their own physician. Although referral from (a trusted) family physician may increase racial/ethnic diversity, this eligibility criterion poses a barrier to individuals without health insurance [81] or those who either do not have a personal doctor or do not visit medical professionals due to the costs, both of which is common in underrepresented populations [82, 83]. We would recommend researchers offer reimbursement for any incurred costs and provide screening at the study site for those without a personal doctor. Prior research in other areas of medicine has also shown incentives can be effective to promote racial/ethnic diversity [84], although ethical concerns have been raised [85].

Regarding recruitment, trials recruiting at the church, using referrals/word-of-mouth, and television and radio advertising had relatively high racial/ethnic diversity levels compared to other recruitment strategies. This is in line with a recent prospective cohort study among NL Black participants demonstrating the effectiveness of recruitment via word-of-mouth/snowballing [64]. Although (mass) mailings (41%), newspapers (25%), and flyers (20%) were frequently used, these passive modes of recruitment are less effective in enrolling those with low socioeconomic status [9] due to factors such as mistrust in research [45] and participants’ limited knowledge of research processes. For example, one study highlighted participants fear of being treated as “guinea pigs,” and the belief research institutions “pry on vulnerable populations” [86]. To address these barriers, efforts to establish collaborations with community organizations or key persons are vital [87], as well as having a diverse research team [86]. Proactive recruitment strategies using a community engaged research approach including community outreach activities at locations relevant to the community may be beneficial – e.g., in-person presentations, face-to-face contact, health events [64]. This is reflected in the studies that recruited at a church (although sample sizes were small), yielding a relatively more diverse sample. Research shows that African American faith institutions can influence health behaviors through their outreach services and special events and by using co-created religiously tailored tools, such as sermon guides [88]. To further promote inclusion, funders might revise their policies to facilitate community organizations as research partners, such as by reducing the load and complexity of documentation processes or improving logistics/infrastructure.

Some studies only recruited participants close to a university or study site, which may inadvertently exclude certain groups from participating, either through negative perceptions among some demographic groups or inaccessibility for those living in rural areas, where the share of Latino individuals is increasing [89]. Several ways exist of improving access [90], e.g., by reimbursing participants for travel or provide transportation services, as well as by testing participants or providing the intervention at home [14, 91–97]. It may be necessary to examine other potential facilitators to trial participation for diverse populations, such as participating outside of office hours. To gather more evidence on recruitment strategies, all future studies should ideally report on the effectiveness of their recruitment strategies by racial/ethnic diversity (see also [17]). Investing in sustained, long-term community partnerships, allocating resources and time to the dissemination of research findings, and actively involving community members in the design process can play a crucial role in improving feasibility, recruitment, and retention [64].

This review has both strengths and limitations. It provides a clear view of current practices in dementia prevention trials in the USA and the possible effects of eligibility criteria and recruitment strategies. A limitation is the small (and likely selective) number of studies reporting on racial/ethnic diversity. Second, the eligibility criteria were extracted from publications as well as registries; the descriptions of these criteria may have been more elaborate in the study protocols that are not publicly available. Third, racial/ethnic diversity was defined in % White participants and not split by racial/ethnic group.

This review has provided a first overview of the eligibility criteria and recruitment strategies used in dementia prevention trials in the USA, in light of participant racial/ethnic diversity. Although we highlighted several medical conditions and recruitment strategies that are associated with participant racial/ethnic diversity, a full understanding of their impact remains elusive. No one exclusionary criterion appears to inhibit diverse participation. Rather, it appears that many criteria, and the continued structural barriers and systemic racism long fostered by the medical research enterprise continue to inhibit full representation in dementia prevention trials. Further, reporting of racial/ethnic diversity data must improve – presenting numbers of excluded participants by racial/ethnic diversity for each criterion, as well as the demographics of participants successfully recruited using different recruitment methods.

## Supplementary Material

Supplementary Box 1

Supplementary T2

Supplementary T1

Supplementary T3

## Figures and Tables

**Table 1.** Median %NL White participants for studies that did versus did not use an eligibility criterion

Criterion	Used as criterion, median % NL White participants (*n* trials)^a^	Not used as criterion, median % NL White participants (*n* trials)^b^	Hodges-Lehmann estimator [95% CI]^c^
Health-related criteria and supplements			
Supplement use	95.5% (5/11)	82.5% (19/33)	−9.1 [−22.1; 2.0]
Use of medication	91.0% (10/22)	83.1% (14/22)	−1.5 [−13.2; 11.4]
Gastro-intestinal and/or liver disease	91.0% (6/12)	81.4% (18/32)	−9.2 [−22.1; 1.5]
Laboratory abnormalities	88.1% (5/10)	84.9% (19/34)	1.4 [−13.8; 18.5]
Specified neurological disorder	86.5% (16/28)	83.7% (8/16)	−2.5 [−16.2; 8.1]
Cerebrovascular disease	86.5% (12/23)	83.7% (12/21)	−1.1 [−12.8; 8.1]
Renal disease	85.9% (9/14)	84.9% (15/30)	−1.1 [−11.6; 10.3]
Alcohol abuse	85.4% (8/13)	87.1% (16/31)	4.2 [−10.6; 11.4]
Drug abuse	84.9% (7/12)	91.7% (17/32)	6.9 [−9.2; 12.4]
Visual impairment	84.2% (8/13)	86.5% (16/31)	1.5 [−10.4; 13.0]
Physical/motor impairment	83.1% (7/13)	87.0% (18/31)	2.6 [−11.4; 12.6]
Specified psychiatric disorder	82.5% (6/11)	85.9% (19/33)	3.6 [−11.2; 16.3]
Recent surgery or hospitalization	82.5% (7/10)	85.9% (17/34)	−2.6 [−14.0; 10.6]
Blood-related diseases	82.5% (5/7)	85.9% (19/37)	1.5 [−13.1; 14.5]
Cardiovascular disease	81.4% (13/27)	94.2% (11/17)	11.6 [1.5; 21.0]
Cancer	81.4% (13/20)	91.7% (11/24)	3.3 [−8.1; 14.6]
Diabetes	81.4% (9/18)	91.7% (15/26)	7.1 [−4.6; 16.2]
Sedentary lifestyle	81.4% (7/14)	91.7% (17/30)	9.2 [−3.4; 18.2]
Reduced life expectancy	81.4% (10/11)	92.3% (14/33)	8.0 [−3.8; 15.6]
Chemotherapy or radiation	81.4% (7/7)	91.7% (17/37)	8.1 [−9.2; 15.6]
Weight/BMI	80.0% (4/7)	85.4% (20/37)	6.0 [−11.5; 24.3]
Level of physical fitness	78.7% (4/5)	87.0% (20/39)	4.9 [−10.2; 18.6]
Pulmonary disease	76.0% (7/9)	92.9% (17/35)	12.2 [0.6; 20.4]
Any medical condition that interferes with intervention	76.0% (7/13)	88.1% (17/31)	8.9 [−3.3; 21.0]
Hearing impairment	71.4% (4/6)	86.0% (20/38)	18.1 [5.4; 24.2]

Cognition-related criteria			
Other objective instrument	92.9% (6/8)	83.7% (18/36)	−4.1 [−17.4; 5.4]
MMSE	81.9% (8/14)	87.0% (16/30)	0.3 [−10.4; 13.0]

Other criteria			
Motivation to participate and complete	94.3% (10/15)	82.0% (14/29)	−7.3 [−15.6; 2.0]
Contra-indication to participation	94.2% (6/13)	83.7% (18/31)	−3.4 [−16.8; 8.4]
(English) language proficiency	91.7% (11/16)	81.4% (13/28)	−6.6 [−18.6; 3.0]
Participation in another study	85.3% (6/7)	85.4% (18/37)	−1.0 [−13.2; 11.7]
Informed consent	84.9% (7/13)	85.9% (17/31)	−1.0 [−11.6; 12.0]
Access to study center; no logistical barriers to communication	80.5% (4/5)	87.0% (20/39)	2.4 [−12.8; 18.4]

BMI, body mass index; NL, Non-Latino; MMSE, Mini-Mental State Examination. The following variables are not included in the table because <4 studies used such a criterion and reported on racial/ethnic diversity: “unspecified neurological disorder,” “brain/head trauma,” “unspecified psychiatric disorder,” “endocrine/auto-immune disease,” “headache,” “post-menopausal,” “pregnancy,” “vitamin/mineral deficiency,” “infections/infectious diseases,” “cigarette smoking,” “recent falls,” “recent fracture,” “subjective reporting of cognitive impairment,” “living situation,” “travel/moving during study,” “partner-related criteria,” “ethnicity,” “Spanish language,” agreeing with randomization,” “education/literacy requirement,” “criminal background,” “specified, non-medical situation”.

a Data are displayed as median % White participants (number of studies that reported on race or ethnicity followed after the slash by the total number of trials using this criterion).

b Data are displayed as median % White participants (number of studies that reported on race or ethnicity followed after the slash by the total number of trials that did not use this criterion).

c An interval that does not include zero, i.e., an interval that falls fully below or above zero, indicates an effect that is likely statistically significant. Additionally, a wide interval indicates less precision in the estimate of the difference.

**Table 2.** Median %NL White participants for studies that did versus did not use a specific recruitment strategy

Strategy	Used as strategy, median %NL White participants (*n* trials)^a^	Not used as strategy, median % NL White participants (*n* trials)^b^	Hodges-Lehmann estimator [95% CI]^c^
Communication and advertisement			
Mailings	92.9% (11/18)	82.5% (13/26)	−5.3 [−16.2; 3.5]
Flyers	86.5% (6/9)	84.2% (18/35)	−1.3 [−13.8; 9.3]
Newspaper	81.4% (9/11)	91.7% (15/33)	6.8 [−4.1; 16.7]
Television and radio	78.7% (6/6)	92.3% (18/38)	10.0 [−2.4; 20.4]
Referrals and word-of-mouth	77.8% (6/10)	89.9% (18/34)	12.0 [−1.0; 22.7]

Community oriented			
Retirement home	83.1% (6/8)	87.0% (18/36)	1.0 [−13.0; 12.6]
Near university campus	82.5% (5/7)	85.9% (19/37)	−1.0 [−14.0; 12.8]
Health-care related	81.4% (7/8)	91.7% (17/36)	5.9 [−8.4; 15.6]
Church	74.7% (4/7)	89.9% (20/37)	13.1 [−4.1; 21.4]

Databases			
Research related	81.4% (5/9)	85.9% (19/35)	4.6 [−8.9; 19.8]

NL, Non-Latino. Studies often used more than one recruitment method. The following strategies are not included in the table because <4 studies using such a criterion also reported on racial/ethnic diversity: “social media,” “telephone”; “hospital,” “nursing home”; databases that are “health-care-related,” “university-related,” “municipality-related” and unspecified databases.

a Data are displayed as median %white participants. This is followed by the (number of studies that reported on race or ethnicity followed after the slash by the total number of trials using this criterion).

b Data are displayed as median %white participants number of studies that reported on race or ethnicity followed after the slash by the total number of trials that did not use this criterion.

c An interval that does not include zero, i.e., an interval that falls fully below or above zero, indicates an effect that is likely statistically significant. Additionally, a wide interval indicates less precision in the estimate of the difference.

**Table 3.** Recommendations for eligibility criteria and recruitment

Obstacles regarding eligibility criteria	Recommendation
Inadequate reporting of race/ethnicity	Report percentage screened and enrolled for each racial/ethnic group, providing exclusion rates for each group per eligibility criterion
Variable/unspecific definitions of sedentary behavior	1. Report definition of sedentary lifestyle used in the study 2. A consensus meeting is needed how sedentary lifestyle may be best defined in dementia prevention research (Magnon et al. [62])
Exclusion of participants based on poorly defined exclusion criteria (e.g., “illness that requires >1 visit per month to a clinician”)	Evaluate whether eligibility criteria relating to medical conditions are clearly defined and necessary for safety. Examine whether the eligibility criteria strike an adequate balance between safety and the inclusion of a sample representative of the population for whom the intervention is intended
Requiring physician’s preapproval for participations	Provide complimentary medical screening or compensate for any medical expenses incurred as part of the study
Exclusion of participants due to an assumed suboptimal level of motivation or assumed decreased likelihood of completing the trial	Provide a transparent and unbiased operationalization of this criterion (if used at all). Increase (personal) contact with research staff to esnure clear communication and address any challenges/concerns promptly
Use of the Mini-Mental State Examination as an eligibility criterion	1. Apply screening tools that are fair across diverse populations (e.g., Rowland Universal Dementia Assessment Scale) (Storey et al. [63]) 2. Using different cut-offs depending on the participant’s education level and reading level
Language proficiency requirements leading to exclusion of diverse participants	1. Allow participants speaking any language to participate if adapted materials and staff members speaking that language are available. Translate and culturally adapt study materials 2. Ensure the research team itself is linguistically and culturally diverse 3. Ensure information and consent forms are written in language that is easy to understand
Obstacles regarding eligibility criteria	Recommendation
Insufficient reporting of the number of participants that were approached and successfully recruited per recruitment strategy	Track and report inclusion rates per racial/ethnic group for each recruitment strategy. For an illustrative example, see Walker et al. [64]
Recruitment restricted to areas characterized by limited diversity (e.g., close to a university)	Examine alternative recruitment locations and provide outreach and recruitment in familiar locations. For example, consider opening a satellite research center (or consider remote participation) in the community, reduce in-person visits, or provide compensation for travel costs. Examine opportunities for at home assessments or assessment outside of office hours (i.e., provide flexible research times)
Passive recruitment strategies and recruitment through health-care related databases	1. Examine more active recruitment strategies and recruitment via physicians or through snowballing 2. Explicitly ask participants whether they know of any other people who might be interested in participating 3. Prioritize community engagement and building partnerships through culturally relevant approaches, such as working with faith-based organizations 4. Collaborate with communities in the study design phase and in recruitment using community-based participatory research methods
Lack of awareness that dementia can be prevented	Provide information about dementia prevention to diverse communities (in a culture sensitive way) and engage community organizations to provide information about dementia prevention
Hesitance to engage in research or mistrust rooted in historical injustice/maltreatment	1. Collaborate with community partners and key community leaders 2. Ensure sufficient diversity/representation within the research team
Other obstacles	Recommendation
Insufficient funding/resources available to engage in long-term sustained community partnerships	Funding mechanisms should be in place to create lasting partnerships that are in place before and after prevention trial ends (Aranda et al. [13])
Significant documentation/infrastructure load on community partners imposed by funding agencies	Funders may revise their policies to facilitate community partnerships

## Data Availability

The data that support the findings of this study are not publicly available due to data volume and format. They are available from the corresponding author (S.F.) via email (s.franzen@erasmusmc.nl).
