# Supplementary material for: Diversity in United States Dementia Prevention Trials: An Updated Systematic Review of Eligibility Criteria and Recruitment Strategies
Source: Dement Geriatr Cogn Disord. Author manuscript; Available in PMC 2026 Feb 13. (PMC12366532; doi:10.1159/000543905)
Supplement: Supplementary Box 1 [file NIHMS2104383-supplement-Supplementary_Box_1.docx]

**Box 1.** Questions used to determine eligibility by McDougall 2010 [36]:

1. Are you able to hear conversations on the phone?
2. Are you able to comprehend English conversations?
3. Are you able to articulate enough to be understood?
4. Are you able to participate in two-sided conversations?
5. Can you decipher concrete and abstract conversational content?
6. Are you able to make an appointment for follow-up testing?
7. Can you repeat back the appointment time and place?
