# Supplementary material for: Diversity in United States Dementia Prevention Trials: An Updated Systematic Review of Eligibility Criteria and Recruitment Strategies
Source: Dement Geriatr Cogn Disord. Author manuscript; Available in PMC 2026 Feb 13. (PMC12366532; doi:10.1159/000543905)
Supplement: Supplementary T2 [file NIHMS2104383-supplement-Supplementary_T2.docx]

**Supplementary Table 2.** Median %NL White participants for studies that did vs. did not use an eligibility criterion

| Criterion | Used as criterion, median % NL White participants  (n trials)* | Not used as criterion, median % NL White participants  (n trials)** | Mann Whitney U, p-value | Effect size (r) | |
| --- | --- | --- | --- | --- | --- |
| Health-related criteria and supplements |  |  |  |  | |
| Supplement use | 95,5% (5/11) | 82,5% (19/33) | 70.0, p=.12 | .33 | |
| Use of medication | 91,0% (10/22) | 83,1% (14/22) | 79.0, p=.63 | .11 | |
| Gastro-intestinal and/or liver disease | 91,0% (6/12) | 81,4% (18/32) | 81.0, p=.08 | .37 | |
| Lab abnormalities | 88,1% (5/10) | 84,9% (19/34) | 43.0, p=.78 | -.07 | |
| Specified neurological disorder  *Cerebrovascular disease* | 86,5% (16/28)  86,5% (12/23) | 83,7% (8/16)  83,7% (12/21) | 72.0, p=.65 74.0, p=.93 | .10  .02 | |
| Renal disease | 85,9% (9/14) | 84,9% (15/30) | 72.0, p=.81 | .16 | |
| Alcohol abuse | 85,4% (8/13) | 87,1% (16/31) | 58.0, p=.74 | -.07 | |
| Drug abuse | 84,9% (7/12) | 91,7% (17/32) | 46.0, p=.42 | -.17 | |
| Visual impairment | 84,2% (8/13) | 86,5% (16/31) | 59.0, p=.79 | -.06 | |
| Physical/motor impairment | 83,1% (7/13) | 87,0% (18/31) | 46.0, p=.63 | -.11 | |
| Specified psychiatric disorder | 82,5% (6/11) | 85,9% (19/33) | 43.0, p=.78 | -.07 | |
| Recent surgery or hospitalization | 82.5% (7/10) | 85.9% (17/34) | 72.0, p=.46 | .16 | |
| Blood-related diseases | 82,5% (5/7) | 85,9% (19/37) | 45.0, p=.89 | -.04 | |
| Cardiovascular disease | 81,4% (13/27) | 94,2% (11/17) | 31.0, p=.018 | -.48 | |
| Cancer | 81,4% (13/20) | 91,7% (11/24) | 58.0, p=.46 | -.16 | |
| Diabetes | 81,4% (9/18) | 91,7% (15/26) | 44.0, p=.17 | -.29 | |
| Sedentary lifestyle | 81,4% (7/14) | 91,7% (17/30) | 34.0, p=.11 | -.33 | |
| Reduced life expectancy | 81,4% (10/11) | 92,3% (14/33) | 53.0, p=.34 | -.20 | |
| Chemotherapy or radiation | 81,4% (7/7) | 91,7% (17/37) | 41.0, p=.26 | -.24 | |
| Weight/BMI | 80.0% (4/7) | 85.4% (20/37) | 27.0, p=.35 | -.21 | |
| Level of physical fitness | 78.7% (4/5) | 87.0% (20/39) | 29.0, p=.43 | -.17 | |
| Pulmonary disease | 76,0% (7/9) | 92,9% (17/35) | 27.0, p=.04 | -.42 | |
| Any medical condition that interferes with intervention | 76,0% (7/13) | 88,1% (17/31) | 44.0, p=.35 | -.20 | |
| Hearing impairment | 71.4% (4/6) | 86.0% (20/38) | 11.0, p=.023 | -.46 | |
| Cognition-related criteria |  |  |  |  | |
| Other objective instrument | 92,9% (6/8) | 83,7% (18/36) | 68.0, p=.38 | .19 | |
| MMSE | 81,9% (8/14) | 87,0% (16/30) | 63.0, p=.98 | -.01 | |
| Other criteria |  |  |  |  | |
| Motivation to participate and complete | 94,3% (10/15) | 82,0% (14/29) | 98.0, p=.11 | .33 | |
| Contra-indication to participation | 94,2% (6/13) | 83,7% (18/31) | 69.0, p=.34 | .20 | |
| (English) language proficiency | 91,7% (11/16) | 81,4% (13/28) | 92.0, p=.25 | .24 | |
| Participation in another study | 85,3% (6/7) | 85,4% (18/37) | 58.0, p=.82 | .05 | |
| Informed consent | 84,9% (7/13) | 85,9% (17/31) | 62.0, p=.90 | .03 | |
| Access to study center; no logistical barriers to communication | 80,5% (4/5) | 87,0% (20/39) | 34.0, p=.68 | -.09 | |
| Abbreviations: BMI = Body Mass Index, NL = Non-Latino, MMSE = Mini-Mental State Examination  * Data is displayed as median % white participants (number of studies that reported on race or ethnicity followed after the slash by the total number of trials using this criterion)  ** Data is displayed as median % white participants (number of studies that reported on race or ethnicity followed after the slash by the total number of trials that did not use this criterion)  The following variables are not included in the table because <4 studies used such a criterion and reported on racial/ethnic diversity: ‘unspecified neurological disorder’, ‘brain/head trauma’, ‘unspecified psychiatric disorder’, ‘endocrine/auto-immune disease’, ‘headache’, ‘post-menopausal’, ‘pregnancy’, ‘vitamin/mineral deficiency’, ‘infections/ infectious diseases’, ‘cigarette smoking’, ‘recent falls’, ‘recent fracture’, ‘subjective reporting of cognitive impairment’, ‘living situation’, ‘travel/moving during study’, ‘partner-related criteria’, ‘ ‘ethnicity’, ‘Spanish language’, agreeing with randomization’, ‘education/literacy requirement’, ‘criminal background’, ‘specified, non-medical situation’. | | | | |  |
