# Supplementary material for: Diversity in United States Dementia Prevention Trials: An Updated Systematic Review of Eligibility Criteria and Recruitment Strategies
Source: Dement Geriatr Cogn Disord. Author manuscript; Available in PMC 2026 Feb 13. (PMC12366532; doi:10.1159/000543905)
Supplement: Supplementary T1 [file NIHMS2104383-supplement-Supplementary_T1.docx]

**Supplementary Table 1.** Study characteristics

| Study | Year published | ClinicalTrial.gov or Cochrane Library | Intervention | Reported races - ethnicities in study |
| --- | --- | --- | --- | --- |
| Anderson-Hanley et al. [31] | 2012 | NCT01167400 | Exergaming | - |
| ADAPT research group [25] | 2008 | NCT00007189 | Supplements | White, Black, Latino, unspecified/unknown |
| Assaf et al. [56] | 2016 | NCT00000611 | Diet or nutrition | White, Black, Latino, American-Indian/Alaska Native, Asian, unspecified/unknown |
| Ball et al. [59] | 2002 | Cochrane Library | Cognitive training | White, Black, unspecified/ unknown |
| Barnes et al. [23] | 2013 | NCT00522899 | Cognitive training, Physical exercise | White, unspecified/unknown |
| Boespflug et al. [34] | 2016 | NCT01746303 | Supplements | - |
| Brickman et al. [40] | 2014 | NCT01180127 | Supplements | - |
| Carlson et al. [52] | 2008 | Cochrane Library | Physical exercise | Black, unspecified/unknown |
| Chew et al. [51] | 2015 | NCT00345176 | Supplements | White, Black, American-Indian/Alaska Native, Asian, Hawaiian/Pacific Islander, unspecified/unknown |
| Dekosky et al. [49] | 2008 | NCT00010803 | Supplements | White, unspecified/unknown |
| Dodge et al. [33] | 2008 | Cochrane Library | Supplements | - |
| Espeland et al. [24] | 2014 | - | Physical exercise, Diet or nutrition | White, Black, unspecified/unknown |
| Fragala et al. [57] | 2014 | - | Physical exercise | - |
| Grodstein et al. [99] | 2013 | NCT00270647 | Supplements | - |
| Grodstein et al. [100] | 2007 | NCT00270647 | Supplements | - |
| Henderson et al. [47] | 2012 | NCT00118846 | Supplements | White, Black, Latino, Asian, unspecified/unknown |
| Hernandez et al. [42] | 2018 | NCT00183014 | Physical exercise | Latino |
| Kang et al. [54] | 2009 | - | Supplements | White, Black, Latino, Asian, unspecified/unknown |
| Kang et al. [55] | 2008 | Cochrane Library | Supplements | - |
| Kang et al. [62] | 2006 | - | Supplements | - |
| Lachman et al. [32] | 2006 | Cochrane Library | Physical exercise | White, unspecified/unknown |
| Leckie et al. [29] | 2014 | Cochrane Library | Physical exercise | - |
| Lewis et al. [61] | 2014 | NCT01672359 | Supplements | White, Black, Latino |
| LIFE study investigators [43] | 2006 | NCT00116194 | Physical exercise | White, Black, unspecified/unknown |
| Marquez et al. [22] | 2017 | Cochrane Library | Cognitive training, Dancing | Latino |
| McDougall et al. [36] | 2010 | NCT00094731 | Cognitive training | White, Black, Latino |
| Miller et al. [35] | 2018 | NCT01888848 | Diet or nutrition | - |
| Miller et al. [101] | 2013 | - | Exergaming | White, unspecified/unknown |
| Nocera et al. [30] | 2015 | Cochrane Library | Physical exercise | - |
| Oken et al. [45] | 2006 | Cochrane Library | Yoga | White, Black, Asian |
| Rizkalla et al. [60] | 2018 | Cochrane Library | Cognitive training | - |
| Rossom et al. [48] | 2012 | Cochrane Library | Supplements | White, Black, Latino, American-Indian/Alaska Native, Asian, unspecified/unknown |
| Schrager et al. [58] | 2015 | Cochrane Library | Diet or nutrition | - |
| Shumaker et al. [26] | 2003 | Cochrane Library | Supplements | - |
| Sink et al. [37] | 2015 | NCT01072500 | Physical exercise | White, Black, unspecified/unknown |
| Small et al. [50] | 2014 | NCT01963767 | Supplements | White, unspecified/unknown |
| Smiley-Oyen et al. [44] | 2008 | Cochrane Library | Physical exercise | - |
| Smith et al. [39] | 2010 | Cochrane Library | Diet or nutrition | White, unspecified/unknown |
| Smith et al. [53] | 2009 | Cochrane Library | Cognitive training | White, unspecified/unknown |
| Souders et al. [103] | 2017 | Cochrane Library | Cognitive training | White, Black, Latino |
| Taylor-Piliae et al. [38] | 2010 | Cochrane Library | Physical exercise | White, unspecified/unknown |
| Vidoni et al. [41] | 2015 | NCT01129115 | Physical exercise | - |
| Williamson et al. [46] | 2009 | Cochrane Library | Physical exercise | White, Black, Latino, unspecified/unknown |
| Wollinsky et al. [103] | 2013 | NCT01165463 | Cognitive training | White, unspecified/unknown |
