# Supplementary material for: Diversity in United States Dementia Prevention Trials: An Updated Systematic Review of Eligibility Criteria and Recruitment Strategies
Source: Dement Geriatr Cogn Disord. Author manuscript; Available in PMC 2026 Feb 13. (PMC12366532; doi:10.1159/000543905)
Supplement: Supplementary T3 [file NIHMS2104383-supplement-Supplementary_T3.docx]

**Supplementary Table 3.** Median% NL White participants for studies that did vs. did not use a specific recruitment strategy

| Strategy | Used as strategy, median % NL White participants  (n trials) * | Not used as strategy, median % NL White participants  (n trials)** | Mann Whitney U, p-value | Effect size (r) |
| --- | --- | --- | --- | --- |
| Communication and advertisement |  |  |  |  |
| Mailings | 92,9% (11/18) | 82,5% (13/26) | 92.0, p=.25 | .24 |
| Flyers | 86,5% (6/9) | 84,2% (18/35) | 58.0, p=.82 | .05 |
| Newspaper | 81,4% (9/11) | 91,7% (15/33) | 47.0, p=.24 | -.25 |
| Television and radio | 78,7% (6/6) | 92,3% (18/38) | 27.0, p=.08 | -.37 |
| Referrals and word-of-mouth | 77,8% (6/10) | 89,9% (18/34) | 26.0, p=.07 | -.38 |
| Community oriented |  |  |  |  |
| Retirement home | 83,1% (6/8) | 87,0% (18/36) | 51.0, p=.87 | -.04 |
| Near university campus | 82,5% (5/7) | 85,9% (19/37) | 51.0, p=.84 | .05 |
| Health-care related | 81,4% (7/8) | 91,7% (17/36) | 47.0, p=.46 | -.16 |
| Church | 74,7% (4/7) | 89,9% (20/37) | 17.0, p=.08 | -.36 |
| Databases |  |  |  |  |
| Research related | 81,4% (5/9) | 85,9% (19/35) | 33.0, p=.33 | -.21 |
| Abbreviations: NL = Non-Latino  * Data is displayed as median %white participants. This is followed by the (number of studies that reported on race or ethnicity followed after the slash by the total number of trials using this criterion)  ** Data is displayed as median %white participants (number of studies that reported on race or ethnicity followed after the slash by the total number of trials that did not use this criterion)  Note: Studies often used more than one recruitment method. The following strategies are not included in the table because <4 studies using such a criterion also reported on racial/ethnic diversity: ‘social media’, ‘telephone’; ‘hospital’, ‘nursing home’; databases that are ‘health-care-related’, ‘university-related’, ‘municipality-related’ and unspecified databases. | | | | |
